# Standard of prevention for infectious diseases' prevention clinical trials during pandemics: learning lessons for global policies from biomedical HIV prevention clinical trials and a case study of COVID-19

**DOI:** 10.3389/fpubh.2024.1539840

**Published:** 2025-01-21

**Authors:** Moréniké Oluwátóyìn Foláyan, Karine Dubé, Nicaise Ndembi

**Affiliations:** ^1^Department of Child Dental Health, Obafemi Awolowo University, Ile-Ife, Nigeria; ^2^Division of Infectious Diseases and Global Public Health, School of Medicine, University of California, San Diego, La Jolla, CA, United States; ^3^Division of Epidemiology and Prevention, Institute of Human Virology, University of Maryland School of Medicine, Baltimore, MD, United States; ^4^Africa Center for Disease Control and Prevention (Africa CDC), African Union Commission, Addis Ababa, Ethiopia

**Keywords:** standard of prevention, HIV prevention, pandemics, epidemics, outbreaks, infectious diseases, stakeholder engagement, best practices

## Abstract

Lessons from biomedical HIV prevention research indicate that standard of prevention packages evolve over time, and require active engagement of stakeholders and community advocates to define packages accept to community members and trial participants. Using COVID-19 prevention research as an example, this paper discusses the reasons why a standard of prevention package must be defined for infectious diseases prevention research, what the minimum content of this package may be, the importance of stakeholder engagement in defining the package, the role of the government, and ethical considerations. As the experience from the HIV pandemic had shown, multiple ethics guidelines argue for a comprehensive standard of prevention package for biomedical HIV prevention trials that does not preclude the inclusion of newly developed HIV prevention tools including those experimental products listed for emergency use during health crisis. In the case of COVID-19, the standard of prevention package should include at a minimum, risk reduction counseling on physical distancing, provision of hand sanitizers, education on how to use available prevention tools, and provision for the possibility of vaccine-induced seropositivity. When pre-exposure prophylaxis studies are conducted for healthcare workers and home carers, personal protective equipment should be provided. Regional and country level regulatory provisions on these issues can provide critical guidance for research design and implementation.

## Introduction

The standard of prevention for biomedical HIV prevention clinical trials is an extensively negotiated package that evolved as new HIV prevention technologies became available (1). Civil society organizations were at the forefront of the research agenda advocating for packages that prioritized study participants' safety. Community advocates were concerned about differing standards of prevention for clinical trials conducted in resource-limited vs. resource-rich countries (2), and the inadequacy of prevention packages in prior or ongoing trials (3). The current standard of prevention package for biomedical HIV prevention clinical trials include provision of internal and external condoms and water-based lubricants, HIV testing and counseling, safer sex and risk reduction counseling, treatment of sexually transmitted infections, education and provision of or referral for voluntary male circumcision, and access to sterile injecting equipment and pre-exposure prophylaxis (PrEP) (4). Advocates also argued that persons who seroconverted during trials are referred to accessible HIV management since this is critical to achieve better HIV outcomes (5, 6).

Table 1 provides a summary of paradigmatic biomedical HIV prevention trials that community advocates had engaged with in the early days of PrEP trials (1996–2016), and the changes in the standard of prevention packages that resulted from these engagements. These include concerns with the continued use of placebo arms in prevention of mother-to-child-transmission trials (2), access to risk reduction counseling to address preventive misconception (7, 8), provision of female condoms for women (3), and access to sterile injecting equipment for people who inject drugs in addition to condoms (9). More recently, advocates had to agitate for the inclusion of HIV PrEP as a standard of prevention in HIV prevention trials, even in countries where PrEP was not part of the HIV prevention regimen (4, 10)—this despite ethics guidelines stipulating that study participants should have “*access to all state of the art HIV risk reduction methods… throughout the duration of the biomedical HIV prevention trial* *(**11**);* and that *new HIV risk-reduction method should be added, based on consultation among all research stakeholders including the community, as they are scientifically validated or as they are approved by relevant authorities”* (11). The arguments against inclusion of PrEP for HIV in vaccine research was due to increase in trial costs resulting from an increase in the sample size, and concerns with scientific validity of the trial (9). In spite of these argument, the persistence of advocates paid off and a HIV vaccine trial, inclusive of PrEP, was designed (12).

**Table 1:** Example of biomedical HIV prevention trials with which advocates engaged with to negotiate HIV prevention packages.

**Trial**	**Brief on trial**	**Controversy**	**Outcome**
HIVNET 012 (13)	A 1997 study conducted in Uganda to explore if a single dose of nevirapine given to both mother and baby was a very safe and effective way to prevent the mother to child transmission of HIV.	The study continued to enroll study participants 14 months after Zidovudine was shown to reduce HIV-1 transmission; and 6 months after the World Health Organization recommended its use ante- and intra-partum in developing countries (14)	Revision of ethics guidelines to stipulate that when there is an effective therapy, a placebo trial is unethical (10). A superiority or non-inferiority study design is what is appropriate
Nonoxynol-9 (15)	Nonoxynol-9 gel, a spermicide, was used in a Phase 3 clinical trial to evaluate the prevention of HIV infection through the vagina. The study started in 1996 (15).	The study showed that there was increased risk for HIV infection using Nonoxynol-9 gel. The issue of therapeutic misconception was raised and the argument for risk reduction counseling was made	UNAIDS and WHO documents that included risk reduction counseling as a standard of prevention (16, 17)
Early pre-exposure prophylaxis trials	The 2004/2005 trials planned to evaluate the efficacy and safety of Truvada as a pre-exposure prophylaxis. Study participants included sex workers in Cambodia, Cameroon and Nigeria, and people who inject drugs in Thailand (6, 17)	Concerns were raised about the absence of female condoms in the prevention package for female sex workers, and no plans for access to sterile needles for people who infected drugs. There was also concern about poor plans for management of persons who sero-converted for HIV during the trial (3).	Community engagement in the design and implementation of biomedical HIV trials was considered an ethical imperative for research; and documents were developed (17) and revised (18) to highlight this.
HVTN 505 (19)	A phase 2b trial, testing two HIV vaccine candidates designed to elicit antibody and T-cell responses, using a DNA prime and rAd5 vector boost regimen. It enrolled high-risk, HIV-negative participants, focusing on protection against HIV acquisition, viral load set-point, and vaccine safety. The trial started in 2009 (19)	Concerns were raised because three PrEP trials showed efficacy in 2010 and 2011 (20–22) and the FDA approved Gilead's Truvada^®^ for HIV prevention in specific populations in 2012; event that all happened after the study started recruiting (10).	Community consultations led to the decision to educate participants about PrEP, refer participants wanting access to PrEP providers, and had Gilead donate product for study participants (10).
HVTN 702 (23)	A Phase 2b/3 trial conducted in South Africa that evaluated the efficacy, safety, and tolerability of ALVAC-HIV (vCP2438) + bivalent Subtype C gp120/MF59 in HIV-seronegative South African adults. The study started in 2016 (24)	Concerns were raised that the status of PrEP access in the general public had improved since HVTN 502; and it was a HIV prevention tool in South Africa (25)	A PrEP fund was set up, Truvada was donated by Gilead for the trial, a fund manager appointed and the research promotional and educational materials were updated.

Since then, HIV prevention clinical trials have included newly developed HIV prevention tools in the clinical trial protocols, including amending study protocols to accommodate new HIV prevention tools as they are developed. These include the FRESH study on HIV acquisition and acute infection, aiming to identify biological risk factors and address gaps in vaccine and cure research (26) that changed its study protocol to offering PrEP through the trial instead of referring for access; the Antibody Mediated Prevention (AMP) trials for broadly neutralizing antibodies (bNAbs) accounted for PrEP in their design and assessed participants' use of PrEP alongside the investigational intervention (27), and the Evidence for Contraceptive Options and HIV Outcomes (ECHO) study that adapted their protocols to ensure comprehensive HIV prevention counseling, including PrEP, for participants (28).

The argument for a comprehensive HIV standard of prevention package provided by the research team for study participants was based on the ethical principle of beneficence and non-maleficence. Study participants had the right of access to a global standard of prevention irrespective of their country of residence; and the right to reduced exposure to prevent harm while investing themselves for a global good (4). Biomedical HIV prevention ethics guidelines request for research-community dialogue to negotiate the inclusion of newly validated HIV prevention modalities as part of HIV prevention packages (11) in recognition that stakeholder engagement with research design, implementation, monitoring and results dissemination is an ethical imperative (29). However, the quality of many of these engagement in resource-limited setting has been poor when it holds (30, 31).

Multiple infectious diseases have emerged as a global threat since HIV. These include Ebola, Zika, dengue, Middle East respiratory syndrome, severe acute respiratory syndrome, influenza (32), COVID-19 in 2019 (33), and more recently, mpox in 2022 and 2024 (34). There could be many other such emerging infectious diseases threats in the future (35). In response, prevention clinical trials—vaccine, PrEP, post exposure prophylaxis products—would be planned. Often, because these are emergencies, there are often no organized community advocacy nor community engagement plans with competency in the disease of interest, to guide the design and implementation of these prevention trials. The implication is that the community concerns may not be taken into consideration when defining the standard of prevention packages for infectious diseases' clinical trials conducted during the outbreaks or emergencies. This increases the risk for variability in the prevention package across research sites and countries based on the requirements made by research ethics committees. Yet, the variability in prevention package across research sites and countries has implications for the comparability of trial results. Some of the variations observed in the standards of HIV prevention packages in the past may be linked to ambiguities in ethics guidelines. Different ethics guidelines set different norms for standards of prevention (36) highlighting the need for consensus in the field.

While HIV prevention research offers valuable lessons, it's crucial to recognize that new infectious disease outbreaks may have distinct modes of transmission, virology, and sociocultural impacts, necessitating the need to tailor prevention strategies. We build on the HIV experience by identifying common ethical and practical frameworks, such as the need for community engagement, informed consent, and stakeholder involvement in developing prevention packages. The minimum content for such packages should include clear, context-specific strategies for prevention, treatment access, and continuous community feedback to ensure relevance and effectiveness in diverse contexts.

In this paper, we discuss the reasons why a standard of prevention package needs to be defined for infectious diseases' prevention research using COVID-19 as a case study, we highlight what the minimum content of this package may be, the importance of community and stakeholder engagement in defining this package based on lessons learned from biomedical HIV prevention research, and ethical considerations for defining this package. In addition, we also discuss the roles of governments and policy-makers in defining and ensuring the inclusion of prevention packages in clinical trials of infectious diseases as exemplified by HIV. We conclude with initial considerations on how to fast-track the process of defining and refining over time, the standard of prevention package for infectious diseases' clinical trials conducted during outbreaks, epidemics and/or pandemics.

## Main text

A defined standard of prevention package for infectious diseases prevention trials provided to all participants in clinical trials is important as this helps minimize the risk of infection and reduces the potential for undue harm (4). This has implications for participant welfare, scientific validity and efficiency of the trial, framing of the research question, and is relevant for health policy decision-making (37). Engaging stakeholders to define the standard of prevention for infectious diseases' prevention trials has multiple values, which includes reducing the risk for negative publicity about the trials (38), preventing trials disruption (6), and facilitating community education that can dispel existing and emerging myths and misconceptions about vaccine research, including concerns about the safety of a fast-tracked vaccine development process (39–41).

Regarding the ethics guidelines on standards for research during the COVID-19 pandemic, the World Health Organization (WHO) highlighted the need for “*fair and meaningful community engagement and inclusive decision-making*” in the design, implementation, and evaluation of the research (42). The guidance document for managing ethical issues in infectious disease outbreaks also recommend that community should be involved in discussions about the acceptability of the study methodology (43). The Good Participatory Practice Guidelines for Trials of Emerging (and Re-Emerging) Pathogens (GPP-EP) builds on the guidance document, provides details on how to meaningfully engage communities, and requires that the *best-proven standard of prevention refers to the package of comprehensive state-of-the-art information and tools provided or made available to participants in an emerging pathogen prevention trial. The locally available standard of prevention in an emergency setting may be lower than the best-proven global standard. Determining what level of prevention a trial will offer requires deliberation with relevant stakeholders, including both women and men [all genders], about how best to achieve the highest level possible and what ethical justifications are required to support a trial providing a higher standard or a lower one that is aligned with that available to others in the population* (44).

Based on current understandings of pandemics, the standard of prevention package for infectious diseases prevention trials (vaccines, pre- and post-exposure prophylaxis) for infectious diseases such as COVID-19, should include at the minimum, risk reduction counseling and hygiene practices, provision of hand sanitizers (when applicable), and education on engagement in public places. When dealing with respiratory diseases, the provision of face masks to study participants and counseling on physical distancing should also be included (45). Participants also need to be informed on the possibility of a vaccine-induced seropositivity (46). When PrEP studies are conducted for healthcare workers and home carers, personal protective equipment should be provided with training on how to don the equipment (47). Decisions also need to be made on the frequency of conducting testing during trial participation, diagnostic tools and testing algorithms.

It is also important to discuss with stakeholders, the possible implications for vaccine research if and when a PrEP product is developed; and to discuss treatment access for study participants who contract the infection as trial participants (with consideration for third party access to prevention and care due to infectivity). Whatever consensus are reached on the standard of prevention for a trial, there should be the caveat that the package will be reviewed as new scientific information emerges: public knowledge will evolve quickly and trial designs should remain open and flexible to emerging information and technologies for updating standards of prevention over time.

Many researchers may be minimalistic, and therefore argue for the barest minimum as the standard of prevention though a few research have taken their ethical responsibilities seriously and even exceed standard of prevention requirements in ethics guidelines during a number of HIV prevention research (48, 49). Arguments that were ushered for the exclusion of PrEP from HIV vaccine research, and may be similarly argued for the exclusion of PrEP in infectious disease vaccine research include: gaining access to PrEP provided at no costs in the trials, there is the possibility of undue inducement, and enough to make participants want to continue in a risky study for PrEP access purposes (10). PrEP has not been widely implemented and it is not a standard of practice in any country (10). Additional arguments may be that inclusion of PrEP in the standard of prevention package may significantly enhance or detract from the usefulness of the primary trial results, by lowering the microbial load set-point thereby affecting the scientific integrity of the study (10). Further, PrEP may significantly interact with the vaccine products and affect either safety or efficacy of the study (10). PrEP may also cause side effects that make the interpretation of study results challenging, study participants may have challenges with adherence, and it may cause behavioral disinhibition (10). Other arguments include the additional costs for the study implementation, logistical considerations, and the large sample size required for the study when gold standard prevention products are provided (50).

There are however undeniable ethical reasons for ensuring trial participants have access to a comprehensive package of prevention during prevention clinical trials. The principle of respect for persons recognizes the need to treat trial participants in ways that responsibly recognizes their autonomy, dignity and inherent rights (51, 52). The principles of beneficence and non-maleficence require that efforts be made to meet basic health needs of individuals while minimizing undue harm (51). The principle of justice warrants fairness in ensuring trials are sensitive to persons who are especially vulnerable to harm, and procedural justice in particular ensures the voice of persons affected by the research are included in the decision-making process (53). These principles are still applicable during the COVID-19 era (54). The principle of solidarity justifies the need for researcher-community engagement in defining the standard of prevention package in the face of a common global threat (55). Finally, the principle of reciprocity recognizes the need to make fitting and proportional returns for the contributions study participants make (56, 57).

These ethical concerns are reasons for a number of ethical controversies by community advocates. In the oral tenofovir controversies that ensued regarding the Phase 2/3 tenofovir trial in Nigeria, community activists were concerns include the need for long-term care for participants who test HIV-positive, defining success criteria, skipping Phase 1 trials to progress to Phase 2/3, ensuring informed consent, and the establishment of a Community Advisory Board (58). The follow up documentation of all the community-led agitations about the trial highlighted commonalities across countries: need for community involvement, concerns over informed consent, and the lack of local researcher engagement. Communities also raised issues about the trial's safety data, the rushed timeline, and inadequate communication. There were calls for protocol revisions, better access to treatment, and addressing the ethical review process. Media played a role in shaping public perception, and there was frustration with researchers' reluctance to engage in dialogue, highlighting a need for improved community-researcher relations (3). More recently, in South Africa, activists raised concern about the standard of prevention in the TASK study—a study evaluating whether the BCG vaccine could help protect healthcare workers and staff against serious COVID-19 disease. Activists allege that the trial did not provide standard of prevention to participants. These controversies can be prevented by engaging communities in standard of prevention decisions (58).

## Governance and government oversight for infectious diseases' prevention clinical trials

Government involvement is critical in setting and supporting the implementation of clear ethical guidelines for clinical trials, ensuring that community engagement and stakeholder involvement are integral to trial design and implementation. The International Health Regulations promote countries taking leads for coordinating preparedness and response efforts to health emergencies, ensuring national health systems are robust and capable of handling outbreaks, and fostering international collaboration to enhance the effectiveness of public health interventions (59). The recent mpox outbreak in Africa that led to the declaration of a public health emergency of continental security (60), and the Marburg responses by Rwanda to independently act to procure monoclonal antibodies (MAB) and remdesivir compassionate use to treat those severely ill from Marburg infection (61, 62) are indications for the need for country ethics regulatory guidance on engagement for infectious diseases' prevention clinical trials.

Conducting research during public health emergencies is an ethical obligation (63), requiring clinical trials to be conducted swiftly and rigorously to evaluate the safety and efficacy of unproven interventions, including “off-label” uses (64). However, a rapid, large-scale, and internationally coordinated research response must not compromise the equi provision of standard prevention packages, regardless of an individual's country of residence. These packages should also include emergency access to unproven clinical interventions outside of trials, as agreed upon during health crises.

Decisions on the standard prevention packages for planned infectious disease clinical trials must involve collaboration with community representatives (65) and be informed by lessons learned from past outbreaks (66). National regulatory bodies should provide clear ethical guidelines for designing and implementing preventive clinical trials during emergencies. They must also establish mechanisms to enforce policies ensuring the standardization and equi delivery of prevention packages in infectious disease clinical trials.

Going forward, there is the urgent need for global action, possibly conveyed by the WHO, to define a minimum standard of prevention package for infectious diseases' prevention research, and for ethics committees to learn and screen prevention research protocols for these packages. Ethics committees and Institutional Review Boards also need to request for evidence of stakeholder and community engagement and negotiation for standard of prevention packages for prevention trials as a measure to safeguard against possible disruption of clinical trials that may be of concern to key constituent groups. This was rarely practiced as considerations for implementing the standards of prevention in HIV prevention studies (67). The nuances needed to negotiate the development of a prevention package represents a significant challenge because of the plethora of ethical considerations to address when planning a biomedical HIV prevention trial (68). This does not preclude however, the need to initiate and sustain the dialogue and attempting resolve these considerations by learning from the field of HIV prevention research and other fields.

Ironically, the International Pandemic Preparedness Secretariat does not prioritize the development of PrEP and post exposure prophylaxis products as countermeasures in the first 100 days of a pandemics (69). The focus is on accurate and approved rapid point of care diagnostic tests, initial regimen of therapeutics, and vaccines ready to be produced at scale for global deployment (70). The past pandemic had shown that vaccine development may take time, access is limited, and PrEP and post exposure prophylaxis can bridge the gap.

Renewed advocacy is needed to recognize PrEP products as a priority agenda in any infectious disease crisis that could threaten global security due to the possibility of the re-emergence of the disease as learnt from the current Mpox outbreak—a disease that led to the declaration of a public health emergency of international concern by the WHO in 2022 and 2024 (34).

## Conclusion

The definition of standards of prevention for infectious diseases prevention trials, and the description and implementation of a standard of prevention package for these trials, is essential. The process of defining these standards should follow best participatory and community engagement practices, with attention to the rapidly evolving science in any infectious diseases outbreak. Together, we must draw lessons from the past to safeguard the ethical integrity of infectious disease prevention trials, while also forging a future where the world is protected from the pervasive threat of infectious diseases. Global and regional regulatory governance mechanisms are needed to provide standardized guidance for countries and research teams to make informed decisions especially in the growing phase of the use of experimental infectious disease prevention products during emergencies.
